# MicroRNA-30e-5p Regulates SOCS1 and SOCS3 During Bacterial Infection

**DOI:** 10.3389/fcimb.2020.604016

**Published:** 2021-01-27

**Authors:** Richa Mishra, Pandikannan Krishnamoorthy, Himanshu Kumar

**Affiliations:** ^1^ Laboratory of Immunology and Infectious Disease Biology, Department of Biological Sciences, Indian Institute of Science Education and Research (IISER) Bhopal, Bhopal, India; ^2^ WPI Immunology, Frontier Research Centre, Osaka University, Osaka, Japan

**Keywords:** innate immunity, microRNA, host-pathogen interaction, bacterial infection, host-directed therapy

## Abstract

Host innate immunity is the major player against continuous microbial infection. Various pathogenic bacteria adopt the strategies to evade the immunity and show resistance toward the various established therapies. Despite the advent of many antibiotics for bacterial infections, there is a substantial need for the host-directed therapies (HDTs) to combat the infection. HDTs are recently being adopted to be useful in eradicating intracellular bacterial infection. Changing the innate immune responses of the host cells alters pathogen’s ability to reside inside the cell. MicroRNAs are the small non-coding endogenous molecules and post-transcriptional regulators to target the 3’UTR of the messenger RNA. They are reported to modulate the host’s immune responses during bacterial infections. Exploiting microRNAs as a therapeutic candidate in HDTs upon bacterial infection is still in its infancy. Here, initially, we re-analyzed the publicly available transcriptomic dataset of macrophages, infected with* *different pathogenic bacteria and identified significant genes and microRNAs common to the differential infections. We thus identified and miR-30e-5p, to be upregulated in different bacterial infections which enhances innate immunity to combat bacterial replication by targeting key negative regulators such as *SOCS1* and *SOCS3* of innate immune signaling pathways. Therefore, we propose miR-30e-5p as one of the potential candidates to be considered for additional clinical validation toward HDTs.

## Introduction

Variety of commensal bacteria were considered beneficial to human host and their role is crucial for the host survival. In contrast, several bacteria qualify the category of potential pathogens to cause serious health ailments in humans ranging from the food-borne illnesses caused by species such as *Listeria monocytogenes* and *Salmonella typhi*, as well as tuberculosis caused by *Mycobacterium tuberculosis* and also associated with oncogenesis. Additionally, bacterial infections are associated as a secondary infection to many infectious and non-infectious diseases, which further enhance the severity of primary disease, for example influenza virus and HIV infection. Furthermore, the alarming elevation of the antibacterial resistance against any bacterial disease possess biggest global threat and is a critical cause for the millions of human deaths annually around the world (Laxminarayan et al., 2013). Like viruses, bacteria can also cause outbreak, leading to sever health damage and lives. Recently, a food-borne- bacteria *Listeria monocytogenes* caused an outbreak in South Africa leading to severe illness and deaths among the population (de Noordhout et al., 2014; Allam et al., 2018; Desai et al., 2019; Thomas et al., 2020). Therefore, re-exploring the host factors against bacterial infections might is needed.

Innate immunity is the first line of defense accelerates when a pathogen encounters the host. Host cells express pattern recognizing receptors (PRRs) which sense a diverse range of invading pathogens including bacteria through PAMPs (pathogen associated molecular patterns) and triggers the immune responses which subsequently eliminate the infection (Kawai and Akira, 2010; Kumar et al., 2011). Macrophages are one of the major innate immune cells also termed as professional phagocytes which helps in binding and clearance of the invading bacterial pathogens (Nau et al., 2002). Additionally, non-immune epithelial cells aid in immune activation to challenge the bacterial infection (Francis and Thomas, 1996). However, almost all pathogenic bacteria develop certain mechanisms to manipulate the host immune system for their survival by various immune evasion strategies (Diacovich and Gorvel, 2010; Reddick and Alto, 2014).

The activated immune system may lead to excessive secretion of inflammatory molecules like interferons and pro-inflammatory cytokines. Hence, immune actions are tightly regulated at various levels. One important regulatory factors and fine tuners of immune system were the microRNAs (miRNAs). miRNAs are small non-coding RNAs of length ranging from 18-22 nucleotides in their mature form. They bind to the partially complementary sequences of the 3’untranslated regions (3’-UTR) in mRNA transcript of the gene to inhibit the expression of the corresponding gene at post-transcriptional level. The miRNAs have been previously shown to be involved in the regulation of bacterial infections and also employed by the bacteria for their survival (Izar et al., 2012; Maudet et al., 2014; Das et al., 2016; Zhou et al., 2018). Host directed therapy (HDT) is one of the recently emerging approach against infectious diseases which majorly aims to directly affects the host factors and machineries which play crucial role in the encroachment and survival of the pathogens (Kaufmann et al., 2018). In previous studies, miRNAs recommended for HDT in bacterial infections (Iannaccone et al., 2014; Sabir et al., 2018) but still the approach of considering miRNAs for HDT lies in its infancy.

In present work, we aimed to identify the miRNA-mediated regulation common to wide range of bacterial infection. We initially re-analyzed the RNA-sequencing dataset GSE73502, in which peripheral blood mononuclear cells (PBMCs) of healthy volunteers were differentiated to macrophages then infected with *Listeria monocytogenes* and *Salmonella typhimurium* respectively (Haraga et al., 2008; Pai et al., 2016). Both the bacteria have different genetic composition and varied immune activation mechanisms associated with them to be used as the model bacteria for understanding the host-bacterial interactions (Corr and O’Neill, 2009). We determine high confidence genes (HCGs) using robust rank aggregation method. Then after applied miRNAs-seed enrichment analysis to HCGs, which identified miR-30-5p family as the highly enriched family of miRNAs within the host. Our study proposed the role of miR-30e-5p (miR-30e) in modulating innate immunity during bacterial infections, due to its significant upregulation during pathogenic infections and PAMPs stimulation. Altogether, our finding concludes that miR-30e targets the 3’-UTR of *SOCS1* and SOCS3, crucial negative regulators of innate immunity which enhances the innate immune responses and reduces the bacterial replication of *Listeria monocytogenes* and *Uropathogenic E. coli* – representative of both gram-positive and gram-negative bacterium respectively, causing severe diseases like listeriosis and urinary tract infections. This further proposes that miR-30e might considered as the potential candidate for HDTs during infectious diseases caused by intracellular bacteria.

## Materials and Methods

### Cell Lines, Bacteria, and Reagents

HEK293 human embryonic kidney cells (ATCC CRL-3216), Raw 264.7 (Cell Repository, NCCS, India), HeLa cervical cancer cells (Cell Repository, NCCS, India), were cultured in Dulbecco’s modified Eagle’s medium (DMEM) supplemented with 10% fetal bovine serum (FBS) and 1% Antibiotic-Antimycotic solution. DMEM, FBS and Antibiotic-Antimycotic solution were purchased from Invitrogen. Human PBMCs were isolated from whole blood as reported previously (Ingle et al., 2015). The seeded cells were washed with phosphate-buffered saline (PBS) prior to infection. Then cells were infected in serum-free DMEM/RPMI with *L. mono.* for 2 h and *UPEC* for 1 h with 50 MOI after attaining optical density (OD 600) of 0.4 to 0.8. After infection cells were washed twice with serum free DMEM/RPMI and supplemented with complete DMEM/RPMI and gentamicin (75ug/ml, Sigma) for 24 h at 37°C, 5% CO_2_. Cells were harvested after 24 h in BHI media containing 1X Triton (Thermo Scientific) and/or Trizol (Ambion Life Tech.) for CFU assay and mRNA quantification. For electroporation of human PBMCs, 1 X 10^6^ cells were suspended in Opti-MEM (Invitrogen) containing 50 nM mirVana miRNA mimics (Ambion). The cells were pulsed twice with 1000 V for 0.5 ms with a pulse interval of 5 s with the Gene Pulser Xcell electroporation system. The cells were then transferred to RPMI supplemented with 10% FBS. Then infected with *L. mono.* with 50 MOI. Transfection of HeLa cells with miRNA mimics, inhibitors and control mimics/inhibitors and/or plasmids was performed with Lipofectamine 2000 or 3000 (Invitrogen) according to the manufacturer’s protocol. Stimulation of cells was carried out using LPS and CpG from Sigma and Invivogen. DMEM, FBS, Opti-MEM, RPMI, and Lipofectamine 2000/3000 were purchased from Invitrogen. The miR-30e mimic (miR-30e) (Invitrogen: Catalog number#4464066) or a nonspecific miRNA negative control#1 (miR-NC1) (Invitrogen: Catalog number#4464058) was used according to the manufacturer’s instructions (Applied Biosystems). The miR-30e inhibitor (AmiR-30e) (Invitrogen: Catalog number#4464066) was used to inhibit miR-30e expression in transfected cells.

### Bacterial Infection

*Listeria monocytogenes* (*L. mono.*), a gram-positive bacterium was used for infection (MTCC-1143). Bacteria were grown to the logarithmic growth phase in brain heart infusion BHI (HiVeg™ Media, HIMEDIA) at 37°C with continuous shaking at 200 rpm overnight. Secondary culture was established until desired OD. Bacteria were subsequently washed with fresh BHI and PBS by two steps of centrifugation at (4,000 rpm, 5 min) and diluted in serum free DMEM/RPMI at 50 MOI for infection. Secondly, *Uropathogenic E. coli* (*UPEC*), a gram-negative bacterium was used for infection, *UPEC* bacteria used in the study was GFP-tagged, GFP was induced by using inducing agent IPTG (Isopropyl ß-D-1-thiogalactopyranoside) at a secondary culture without shaking the inoculated tube/s. After obtaining optimal OD, respective bacterial cultures were used to infect the mammalian HeLa cells and PBMCs accordingly. Cell were then harvested to quantify the bacterial population by performing colony forming unit assay and counting the bacterial colonies at different dilutions on BHI plates incubated overnight at 37°C.

### Quantitative Real-Time Reverse Transcription PCR

Total RNA was extracted with the Trizol reagent (Ambion/Invitrogen) and used to synthesize cDNA with the iScript cDNA Synthesis Kit (BioRad, Hercules, CA, USA) according to the manufacturer’s protocol. Gene expression was measured by quantitative real-time PCR using gene-specific primers both for humans and bacteria as analyzed in the results and SYBR Green (Biorad, Hercules, CA, USA) and additionally using 18S and NPM1 (for AGO2-RNA immunoprecipitation experiment) primers for normalization. For quantification of the abundances of miR-30e, real-time PCR analysis was performed with the TaqMan Universal PCR Master Mix (Applied Biosystems) and the miR-30e-5p specific TaqMan miRNA assays. The Taqman U6 assay was used as a reference control. Real time quantification was done using StepOne Plus Real time PCR Systems by Applied BioSystems (Foster City, CA, USA).

### Luciferase Reporter Assays

HEK 293T and HeLa cells (5 X 10^4^) were seeded into a 24-well plate and transiently transfected with 25 nM of mimics (miR-30e and miR-NC1), 50 ng of the transfection control pRL-TK plasmid (*Renilla* luciferase containing plasmid) and 300 ng of the various expression plasmids (containing 3’-UTR of specific genes and *Firefly* luciferase containing plasmid) according to the respective experiments. In another experiment, 300 ng of miR-30e promoter *Firefly* luciferase containing plasmid together with 50 ng of the transfection control pRL-TK plasmid were transfected together and finally infected with *L. mono.* The cells were lysed at 24 h after transfection and/or infection, and finally the luciferase activity in total cell lysates was measured using Glomax machine (Promega, Madison, WI, USA).

### Enzyme-Linked Immunosorbent Assay (ELISA)

HeLa cells were transiently transfected with miR-30e and miR-NC1 and then were infected *L. mono*. bacterial infection then treated with gentamycin. The culture media were harvested 24 h after infection and were analyzed by specific ELISA kits (Becton Dickinson) according to the manufacturer’s instructions to determine the amounts of *IL6* that were secreted by the cells.

### RNA Immunoprecipitations

RNA immunoprecipitations were performed as described previously (Meister et al., 2004; Beitzinger and Meister, 2011). The pIRESneo-Flag/HA Ago2 plasmid was a gift from Professor T. Tuschl (Addgene plasmid #10822). Briefly, HeLa cells transfected with miRNA and infected with *L. mono*. then treated with gentamycin were lysed in 0.5% NP-40, 150 mM KCl, 25 mM tris-glycine (pH 7.5) and incubated with M2 Flag affinity beads (Sigma) overnight. The lysate was then washed with 300 mM NaCl, 50 mM tris-glycine (pH 7.5), 5 mM MgCl_2_, and 0.05% NP-40. The extraction of RNA from the immunoprecipitated RNPs was performed with the Trizol reagent (Ambion, Invitrogen) according to the manufacturer’s protocol.

### Microscopy

HeLa cells were seeded along with cover slips in low confluency and next day transfected with miRNA mimic for 24 h prior to bacterial infection then infected with *UPEC*-GFP for 4 h and treated with gentamycin for 1 h. Afterwards kept in incubator (37°C, 5% CO2) for 24 h. Then cells were fixed with 4% PFA for 15 min at room temperature; permeabilized with 0.05% Triton X-100 in 1 x PBS for 10 min at room temperature; blocked with bovine serum albumin (5 mg/ml) in PBS, 0.04% Tween-20 for 30 min and incubated for 1 h with the relevant primary antibodies diluted in blocking buffer. The cells were then washed three times with PBS and incubated for 1 h with the appropriate secondary antibodies at room temperature. Nuclei were stained with DAPI, phalloidin red was used to stain the actin filaments of the cells. Cover slips then containing cells were carefully mounted on to the glass slides using Fluoroshield (Sigma) as mounting media. Slide was then kept for few hours for drying before imaging. Images were visualized at 40X with Apotome – AXIO fluorescence microscope by Zeiss.

### Re-analysis of the RNA-Seq Dataset

**Figure 1:**
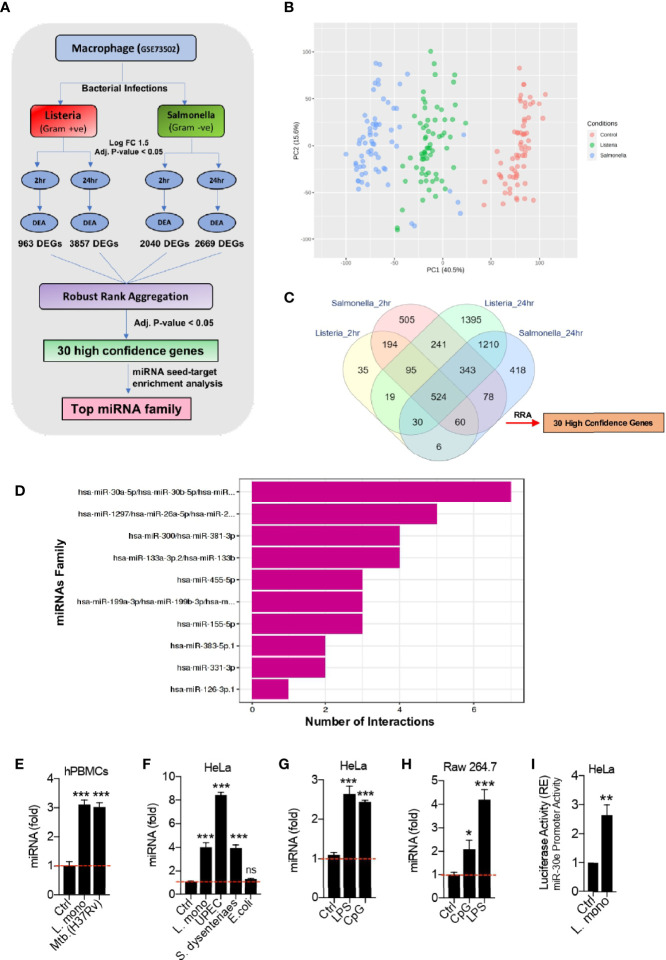
Bioinformatic identification of crucial host genes and microRNAs (miRNAs) associated with bacterial infection in Macrophages. **(A)** Schematic of the bioinformatics pipeline used to identify the high confidence genes and the potential miRNAs that target them, upon infection with two different bacteria at different time points. **(B)** PCA plot shows the segregation of samples between three experimental groups – Control, Listeria and Salmonella infection. **(C)** Venn diagram shows the overlap of differentially expressed genes at 2 h and 24 h of *Listeria* and *Salmonella* infection compared to the corresponding uninfected samples giving 30 high confidence host factors obtained through robust rank aggregation (RRA) method. **(D)** Bar plot showing miRNA seed enrichment analysis for significant high confidence genes obtained through robust rank aggregation method. **(E–H)** Quantification (as determined by qRT-PCR analysis) of the fold changes in the abundances of miR-30e as indicated in hPBMCs, HeLa and Raw264.7 cells in presence of respective bacterial pathogens and PAMPs stimulation. **(I)** Quantification of miR-30e promoter activity by luciferase assay as indicated in HeLa cells. Data are mean +/- SEM of triplicate samples from single experiment and are representative of two independent experiments. ***P < 0.001, **P < 0.01 and *P < 0.05 by one-way ANOVA Tukey test and student t-test.

The raw read counts were obtained from GSE73502 (Pai et al., 2016) through GREP2 R package (Mahi et al., 2019) and were TMM (Trimmed mean of M-values) normalized. Differential expression analysis was performed using EdgeR (Robinson et al., 2010) package with a significance cutoff – logFC >1.5 and adjusted *p-value <*0.05. The differential expression analysis was performed for each time point (2hr and 24 hr) and both the bacterial infections separately. In case of robust rank aggregation (RRA) approach (Kolde et al., 2012), the significant differentially expressed genes (DEGs) obtained from each case were ranked using robust rank aggregation R package, which basically ranks and aggregate the high confidence genes across each list. The high confidence genes were calculated based on the *p-value* adjusted using Bonferroni correction and 30 genes were obtained below the cutoff less than 0.05. In next approach, miRNA seed enrichment analysis was performed using the tool Mienturnet (Licursi et al., 2019). In this, the high confidence genes were used as input for the miRNA enrichment analysis. The number of miRNAs which were predicted to be binding with the high confidence genes were represented using the bar plot (**Figure 1D**). All the analysis was performed in R 3.6 environment and the tool Networkanalyst (Zhou et al., 2019) was used for the generation of PCA plot and Venn diagram.

### Statistical Analysis

All experiments were carried out along with the appropriate controls, indicated as control cells (Ctrl) or uninfected/non-infected cells. Experiments were performed in duplicates or triplicates for at least two or three times independently. GraphPad Prism 8.0 (GraphPad Software, La Jolla, CA, USA) was used for statistical analysis. The differences between two groups were compared by using an unpaired two-tailed Student’s t-test. While the differences between three groups or more were compared by using analysis of variance (ANOVA) with Tukey test. Differences were considered to be statistically significant when *P < 0.05*. Statistical significance in the figures is indicated as follows: ***P < 0.001, **P < 0.01, *P < 0.05; ns, not significant.

## Results

### Bioinformatic Prediction of Host Genes and Their Regulatory MicroRNAs During Bacterial Infection

To investigate the microRNAs (miRNAs) and their target(s) involved in bacterial infection, we robustly re-analyzed the publicly available transcriptomic dataset GSE73502 in Gene Expression Omnibus (GEO) database. The dataset comprises of human macrophages infected with live gram-positive *Listeria* or gram-negative *Salmonella* bacteria, respectively (Pai et al., 2016). We first identified high confidence genes (HGCs) or the host factors from the dataset which were significantly dysregulated upon bacterial infection. Next, we identified the miRNAs targeting these HCGs. The schematic of *in-silico* unbiased pipeline used to perform the analysis is explained in **Figure 1A**. The dataset includes samples of human macrophages infected with bacteria both at early (2 h) and late (24 h) time points that were taken for analysis to cover the maximum number of infected samples during the unbiased analysis as well as to find the wide range host factors involved in crucial cellular machineries both at early and late stages of infection. After normalization, the segregation of different samples was visualized through PCA plot (**Figure 1B**). Differential expression analysis for genes was performed between the non-infected (control) and infected (*Listeria* and *Salmonella*) groups. The 963 and 3857 differentially expressed genes (DEGs) were obtain upon *Listeria* infection at 2 h and 24 h respectively. Similarly, 2040 and 2669 DEGs were obtain upon infection with *Salmonella* at 2 h and 24 h respectively. To determine the significantly dysregulated genes crucial for different cellular responses and common to both bacterial infections at early and late time points, we employed a popular rank aggregation method known as robust rank aggregation (RRA) (Kolde et al., 2012). The overlap of the DEGs were depicted in Venn diagram (**Figure 1C**). Robust rank aggregation method narrowed 30 genes to be significantly dysregulated in all the cases with the adjusted *p-value* of 0.05. These 30 genes or host factors were predominantly related to immune pathways and studied during bacterial infections. In addition, this analysis holds valid presumptions to be taken into consideration as the target cells used here were macrophages from healthy volunteers infected with bacteria (*Listeria* and *Salmonella*), are the crucial innate immune cells to play an important role in defense during bacterial infections. Through this approach we have found 30 HCGs crucial for both the infections. In this connection, we predicted the microRNAs which can modulate these HCGs as the host regulatory molecules proposing their utility in host-directed therapy (HDT). Furthermore, we performed miRNA seed-target enrichment analysis using a tool called Mienturnet (Licursi et al., 2019). This analysis provides the miRNA seed enrichment result from the given query of HCGs. We found the miRNA-seed of miR-30-5p family to be significantly enriched which targets seven of these 30 HCGs as shown in **Figure 1D**. This family consists of five members (miR-30a to miR-30e) with minor sequence difference and major phylogenetic difference as regulated at different chromosomal location. miR-30a/b/c/d has been extensively studied in relation to bacterial infections and immune evasion strategies as discussed later. However, role of miRNA-30e during bacterial infection is poorly understood. Hence, we further focused on miRNA-30e characterization as the host regulatory microRNA and concluded that miR-30e-5p induced upon different bacterial infections and stimulation with bacterial PAMPs in hPBMCs, HeLa and Raw264.7 cells respectively (**Figures 1E–H**). In addition, we estimated the miR-30e promoter activity in presence of *Listeria monocytogenes* (*L. mono.*) infection (**Figure 1I**) and found that bacterial infection controls miR-30e transcriptional regulation to regulate its expression.

### The miRNA-30e-5p Targets Innate Immunity Regulators *SOCS1* and *SOCS3*

**Figure 2:**
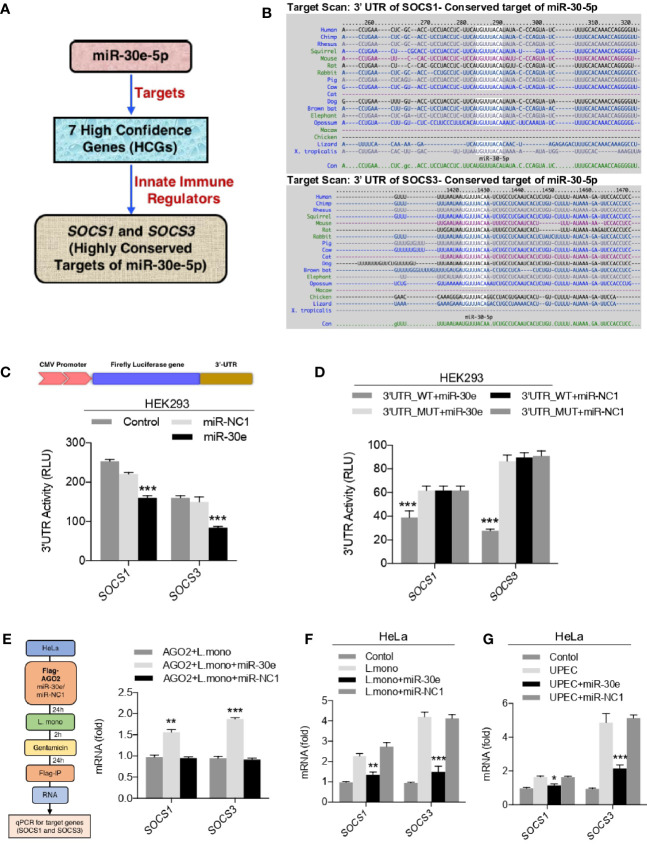
miR-30e-5p targets *SOCS1* and *SOCS3*. **(A)** Schematic of miR-30e-5p targeting of innate immune regulators. **(B)** miR-30-5p binding 3’ UTR site conservation of *SOCS1* and *SOCS3* among wide range of species. **(C, D)** HEK293 cells were transfected with 50 ng of pRL-TK and 300 ng of 3’UTR_WT or 300 ng of 3’UTR_MUT (of indicated genes) together with 25 nM miR-30e or miR-NC1 mimics, 24 h after transfection, the cell was lysed and subjected to luciferase assay. **(E)** Schematic for RNA-immunoprecipitation assay. HeLa cells were transfected with plasmid encoding Flag-AGO2 in presence of miR-30e (50 nM) and miR-NC1 (50 nM) and then infected with *L. mono.* (50 MOI). Serum-free media of cells were replaced after 2 h with complete media containing gentamicin. After 24 h cells were subjected to RNA immunoprecipitation and quantified for *SOCS1* and *SOCS3* transcripts. **(F, G)** Quantification of the fold changes by qRT-PCR analysis in the relative abundances of *SOCS1* and *SOCS3* after infection of **(F)** *L. mono.* (50 MOI) and **(G)** *UPEC* (50 MOI) for 24 h in HeLa cells prior to transfected with miR-30e or miR-NC1 as indicated. Data are mean +/- SEM of triplicate samples from single experiment and are representative of two **(E)** and three **(C, D, F** and **G)** independent experiments. ***P < 0.001 and *P < 0.05 by one-way ANOVA Tukey test.

To demonstrate the post-transcriptional regulation by miR-30e-5p, out of seven enriched host genes, *SOCS1* and *SOCS3* were selected to be analyzed *in-vitro* as shown schematic representation (**Figure 2A**). Because *SOCS1* and *SOCS3*, a key immune negative regulator of PRR-mediated innate immune signaling pathways were obtained after the unbiased miRNA-seed enrichment analysis to demonstrate the role of miR-30e in immune regulation. Notably, they were also observed to be highly conserved targets of miR-30e throughout all the different class of species (**Figure 2B**). Next, to validate the regulation of 3’UTR of mRNA targets, the 3’UTR of the *SOCS1* and *SOCS3* gene were cloned downstream of luciferase gene under the CMV promoter to perform the luciferase assay. It was found that miR-30e significantly reduced the luciferase activity compared to control miR-NC1 (**Figure 2C**). In contrast, introduction of mutation (3’UTR_MUT) in cloned 3’UTR/3’UTR_WT by site directed mutagenesis (SDM) did not change the luciferase activity in presence of miR-30e and it was comparable with 3’UTR_WT (wild type) as shown in **Figure 2D**. miR-NC1 was used as a control for the experiment. Furthermore, we scanned the 3’UTRs of *SOCS1* and *SOCS3* for RNA binding site for AGO2 protein, in CLIP database, which is a key component of the miRNA-mediated silencing known as RNA-induced silencing complex (RISC) and found that the miR-30e make stable complexes with the target genes (Mishra et al., 2020). To validate, miR-30e and negative regulators transcripts *(SOCS1 and SOCS3)* interaction, AGO2 pull-down assay was performed as shown in schematic (**Figure 2E**) and found that introduction of miR-30e significantly enriches the transcript of *SOCS1* and *SOCS3* during *L. mono.* infection compared to the *L. mono.* infection alone or *L. mono.* infection along with control miR-NC1 treated cells, suggesting that miRNA-30e directly interact with the transcript through the formation of RISC. Next, the ectopic expression of miR-30e reduced the expression level of *SOCS1* and *SOCS3* in HeLa cells compared to the control after *L. mono.* infection (**Figure 2F**) and *UPEC* infection (**Figure 2G**). Taken together, miR-30e targets *SOCS1* and *SOCS3* significantly which might regulate innate immunity during bacterial infection.

### The MiRNA-30e-5p Curtails Bacterial Infection by Enhancing Innate Immunity

**Figure 3:**
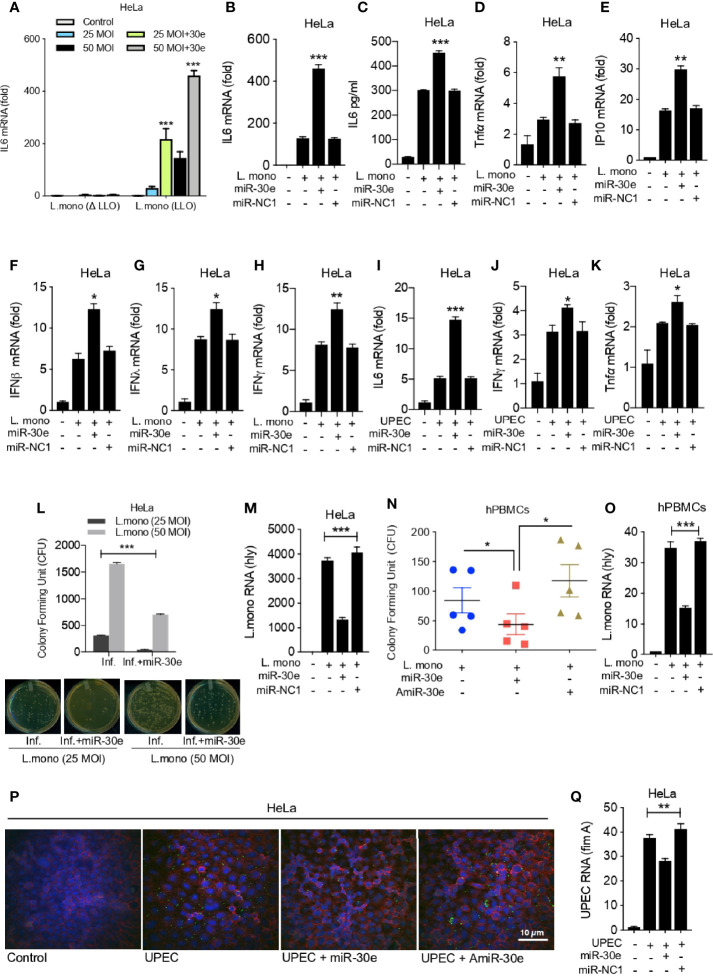
miR-30e-5p elevates innate immunity by inhibiting bacterial replication. Quantification of the fold changes in the relative abundances of respective innate immune transcripts and bacterial transcripts after the 24 h of *L. mono.* infection in the cells transfected with miR-30e (50 nM) or miR-NC1 (50 nM) prior to infection. Expression of *IL6* **(A)** in HeLa cells in presence of listeriolysin O (LLO)-deficient and LLO-sufficient strain of *L. mono*. In HeLa cells at **(B)** mRNA level and **(C)** protein level. Quantification of **(D)** *TNFα*, **(E)** *IP10*, **(F)** *IFNβ*, **(G)** *IFNλ*, **(H)** *IFNy*. Quantification of **(I)** *IL6*, **(J)** *TNFα*, **(K)** *IFNy* after the 24 h of *UPEC* infection in the HeLa cells transfected with miR-30e (50 nM) or miR-NC1 (50 nM) prior to infection. Estimation of *L. mono.* infection in presence of miR-30e by colony forming assay (CFU) in **(L)** HeLa cells and bacterial transcript (*hly*) quantification in **(M)** HeLa cells. **(N)** CFU in hPBMCs. **(O)** Bacterial transcript (*hly*) quantification. Estimation of *UPEC* infection in presence of miR-30e by **(P)** microscopy and **(Q)** bacterial transcript (*fimA*) quantification. Data are mean +/- SEM of triplicate samples from single experiment and are representative of two independent experiments. ***P < 0.001, **P < 0.01 and *P < 0.05 by one-way ANOVA Tukey test.

To understand the physiological implication of miR-30e-mediated targeting of *SOCS1* and *SOCS3*, we investigated innate immune responses and bacterial burden. To this end, we estimated the expression level of innate immune effector genes in presence of *L. mono.* and miR-30e. Notably, absence of listeriolysin O (LLO) or non-pathogenic strain of *Listeria* showed no significant induction of immune response in comparison to LLO containing pathogenic strain of *Listeria* (**Figure 3A**). Therefore, for subsequent study we have used the LLO-sufficient strain. In context to immune regulation upon infection, miR-30e was found to significantly induce the expression of *IL6* both at mRNA and protein levels as quantified by qRT-PCR (**Figure 3B**) and ELISA (**Figure 3C**) in presence of bacterial infection in HeLa cells compared to control miR-NC1. Subsequently, miR-30e also shown to enhance the expression of other pro-inflammatory cytokines and *IFNs* (Type 1,2 and 3) during *L. mono.* (gram-positive) (**Figures 3D–H**) and *UPEC* (*Uropathogenic E. coli:* gram-negative) (**Figures 3I–K**) bacterial infections respectively. Heightened innate immune responses in case of bacterial infections and in presence of miR-30e, prompted us to demonstrate the eligibility of miR-30e-5p toward host-directed therapy parameters. Therefore, we estimated the outcome on bacterial replication in presence of miR-30e. Colony forming units (CFU) assay (**Figure 3L**) and mRNA quantification (**Figure 3M**) of *L. mono. hly* gene in HeLa cells demonstrated that miR-30e reduces the *L. mono.* replication. Similar results were shown in the hPBMCs isolated from five different individuals both in presence of both miR-30e and AmiR-30e (inhibitor of miR-30e) by CFU assay (**Figure 3N**). Quantification of *hly* gene at mRNA levels also demonstrates reduced replication of *L. mono.* in hPBMCs upon miR-30e treatment compared to control miR-NC1 treated cells (**Figure 3O**). Furthermore, microscopy analysis, revealed the reduction in bacterial replication of *UPEC* GFP-tagged gram-negative bacteria in presence of miR-30e compared to AmiR-30e (**Figure 3P**). In addition, quantification of *fimA* virulence gene at mRNA levels demonstrates reduced replication of *UPEC* in HeLa cells upon miR-30e treatment to cells compared to control miR-NC1 treated cells (**Figure 3Q**). Altogether, miR-30e qualifies to combat bacterial replication by enhancing innate immunity *via* targeting *SOCS1* and *SOCS3*, two crucial negative regulators of innate immune signaling cascade during bacterial infections.

## Discussion

The pathogens continuously evolve along the host for their survival and to evade the immune defense mechanisms within the system. Innate immunity delivers protection against various intracellular bacteria by recognition of bacterial PAMPs through various sensors. Several intracellular bacteria infects the host and develop mechanisms to escape from the innate immune activation (Diacovich and Gorvel, 2010). Host-pathogen communication decides the initiation, development and progression of infectious diseases. Majority of hazardous disease like tuberculosis, brucellosis, listeriosis, and salmonellosis are caused by intracellular bacteria (Paul, 2003; Silva, 2012). Listeriosis or commonly known as food-borne disease requires attention in this modern era of differential standards of food supply and reconstruction of food demand strategies. *Listeria monocytogenes* (*L. mono.*) is a gram-positive bacterium causing listeriosis, its pathogenesis being explored in detail and is used extensively as the model for studying different aspects of host-bacteria intricacies. Apart from macrophages, non-immune epithelial cell lines such as Hela cells were also reported to be used in demonstrating infectivity of bacterial infections, particularly, *L. mono.* (Francis and Thomas, 1996). *Uropathogenic E. coli (UPEC)* is another harmful bacteria belongs to gram-negative bacterium and is responsible for causing mild to severe urinary tract infections (UTI) with the ability to counter distinct immune modulation strategies (Olson and Hunstad, 2016). In contrast, evidence suggests, rapid onset of innate immune responses during the clinical manifestations of UTI might provide new biomarkers for the infectious disease (Reygaert, 2014; Ching et al., 2020). An intact and efficiently regulated innate immune environment is critical for host resistance against bacterial infection.

Herein, we re-explored publicly available dataset to perform differential expression analysis. To obtain high confidence genes (HCGs), we used robust rank aggregation (RAA) method to detect the genes that are ranked consistently better than expected under null hypothesis of uncorrelated inputs and assigns a significance score for each gene. Next we performed miRNA seed enrichment analysis for these HCGs, which provided the significant miRNA-target interactions. This approach concluded the identification of mir-30-5p family members to be enriched among the other miRNAs/miRNA-families targeting maximum seven out of 30 high confidence differentially expressed genes (**Figure 1D**). miRNA-30-5p family consists of five members namely miR-30a-5p, miR-30b-5p, miR-30c-5p, miR-30d-5p and miR-30e-5p. miR-30 family has been reviewed and reported to be extensively involved in regulation of development and diseases within the host (Verma et al., 2015; Mao et al., 2018). We selected miR-30e-5p for our study, which is lesser explored (Das et al., 2013; Latorre et al., 2015) to decipher the unknown regulatory mechanism during bacterial infection and subsequent innate immune modulation. In contrast, miR-30a has been shown to be involved in *Streptococcus pneumoniae* and *Mycobacterium tuberculosis* infections (Chen et al., 2015; Wu et al., 2017; Poore et al., 2018). miR-30b and miR-30d has been reported to be associated with *H. pyroli* infection (Tang et al., 2012; Yang et al., 2016). miR-30b/miR-30c, controls the intracellular survival of *Burkholderia pseudomallei* by targeting Rab32 GTPase and regulating phagosome formation (Hu et al., 2019). Seven HCGs those were targeted by miR-30-5p family were *FOSL2*, *GBP2*, *IL2RA*, *SOCS1*, *SOCS3*, *TMEM86A*, *TNIP1*. These factors were reported to be involved in various bacterial infections and other diseases (Cha et al., 2013; Degrandi et al., 2013; Goudy et al., 2013; Muth et al., 2017; Ling et al., 2018; Shamilov and Aneskievich, 2018; De Oyarzabal et al., 2019; Place et al., 2020). SOCS family proteins were considered as the important regulators of inflammatory responses (like interferons and cytokines; *IL6, TNFα*) and being significantly induced by broad range of bacterial infections (Stoiber et al., 2001; Yasukawa et al., 2003; Demirel et al., 2013; Carow and Rottenberg, 2014; Duncan et al., 2017; Alice et al., 2018). Interferons (*IFNs*) and pro-inflammatory cytokines like *IL6* were additionally reported to play crucial roles during Listeria infection that might be connected to SOCS proteins in the Listeria infected system (Stoiber et al., 2001; Hoge et al., 2013; Pitts et al., 2016; Hop et al., 2019). Interestingly, out of these 7 HCGs, *SOCS1* and *SOCS3* were found to be the highly conserved targets of miR-30e with greater binding affinity. Therefore, we sought to characterize miR-30e binding with *SOCS1* and *SOCS3* during bacterial infections.

In the host-directed therapy (HDT), the host factors which are crucial for the infection of pathogen and its survival will be targeted for possible therapeutic outcomes, rather than directly targeting the pathogen, which is true in case of conventional antimicrobial drugs (Zumla et al., 2016; Kaufmann et al., 2018; Baindara, 2019). HDT can help in tackling the anti-microbial resistance which is one of the major challenges in dealing global health concerns. HDT for bacterial infection involves repurposed drugs, synthetic nucleic acids, mono-clonal antibodies, recombinant proteins to enhance the host immune defense against the pathogenic bacterial agent (Wroblewski et al., 2010; Yedery and Jerse, 2015; Bravo-Santano et al., 2019). To interpret the dimension of HDTs *via* microRNA-mediated regulatory mechanism, we thought of understanding the role of miR-30e in innate immune modulation. Hence, we validated the expression of miR-30e, which is upregulated during bacterial infections/PAMPs (**Figures 1E–H**) and selected *SOCS1* and *SOCS3*, important negative regulator of innate immune signaling pathways which are significantly targeted by miR-30e (**Figure 2**). This resulted in reduced bacterial burden and moderate elevation of the innate immune responses within the HeLa cells infected with *L. mono.* and *UPEC* bacteria. On contrary, inhibitor of miR-30e was shown to exhibit opposite effect on bacterial infection (**Figures 3L, N**). Recently, miRNAs were shown as the promising candidates for HDT during many cases of bacterial infections (Iannaccone et al., 2014; Liu et al., 2018; Sabir et al., 2018). miRNAs are the fine tuners of host cellular factors during infectious diseases which reshapes the immunity and inflammatory responses during bacterial infection (Das et al., 2016; Zhou et al., 2018). In conclusion, our findings suggest that miR-30e regulates bacterial infections by interfering with the innate immunity that might challenges the bacterial survival strategy. Therefore, miRNA-30e binding of host factors with emphasis on immune modulation could be exploited as a constructive approach toward HDTs during bacterial infections.

*In vivo* investigation of miRNA-30-5p using mouse model and various bacterial infection system is one of major limitation of this study. These studies will provide mechanistic and physiological parameters more efficiently toward the evaluation of therapeutic potential of this miRNA for the development of HDTs.

## Data Availability Statement

The original contributions presented in the study are included in the article. Further inquiries can be directed to the corresponding author.

## Author Contributions

Conceptualization, RM, and HK. Investigation, RM. Validation, RM. Formal analysis, RM and HK. Data curation, PK. Writing—Original draft, RM and HK. Writing—review and editing, RM, PK, and HK. Supervision, HK. All authors contributed to the article and approved the submitted version.

## Funding

This work was supported by an Intramural Research Grant of IISER, Bhopal, India, to HK. RM is supported by the IISER Bhopal institutional fellowship.

## Conflict of Interests

The authors declare that the research was conducted in the absence of any commercial or financial relationships that could be construed as a potential conflict of interest.
